# Overexpression of IGF2 affects mouse weight and glycolipid metabolism and IGF2 is positively related to macrosomia

**DOI:** 10.3389/fendo.2023.1030453

**Published:** 2023-04-19

**Authors:** Qidi Zhang, Shengtang Qin, Jing Huai, Huixia Yang, Yumei Wei

**Affiliations:** Beijing Key Laboratory of Maternal Fetal Medicine of Gestational Diabetes Mellitus, Department of Obstetrics and Gynecology, Peking University First Hospital, Beijing, China

**Keywords:** insulin-like growth factor 2, mouse model, weight gain, glycolipid metabolism, macrosomia, STAT3/AKT axis

## Abstract

**Objective:**

To investigate the effects of insulin-like growth factor 2 (IGF2) on growth and glycolipid metabolism, as well as the underlying mechanism.

**Methods:**

A mouse model of IGF2 overexpression was constructed to measure weight gain before adulthood, to obtain the values of adult glycolipid metabolism indicators in the peripheral blood and to detect the expression of genes in the IGF2 signaling pathway in different mouse tissues. The present study also explored the independent association between the IGF2 gene and macrosomia by detecting and comparing the expression levels of IGF2 mRNA/H19 RNA in maternal peripheral blood and fetal cord blood of 26 human pregnancies.

**Results:**

In the mouse model, weights of the IGF2-overexpressing mice were significantly higher than those of the control mice at the age of 5-10 weeks. The glucose concentration, total cholesterol and high-density lipoprotein cholesterol (HDL-C) levels of IGF2-overexpressing mice were significantly lower than those of wild-type (WT) mice. Compared with the WT mice, the expression of H19 was significantly decreased in the pancreas and IGF1R was significantly decreased in the muscle of mice with IGF2 overexpression. The expression levels of STAT3 and AKT2 showed significant decrease in liver, muscle and increase in muscle of IGF2-overexpressing mice, respectively. GLUT2 expression showed significant increase in liver, kidney, muscle and decrease in pancreas of mice with IGF2 overexpression. This study also found that in normal mothers with the similar clinical characteristics, IGF2 expression in the maternal peripheral blood and fetal cord blood is an independent factor influencing macrosomia.

**Conclusion:**

IGF2 expression was independently correlated with the occurrence of macrosomia, and overexpression of IGF2 significantly increased the weights of mice at the age of 5-10 weeks and significantly affected the values of adult glycolipid metabolism indicators, which might be the result of changes in the IGF2-IGF1R-STAT3/AKT2-GLUT2/GLUT4 pathway. These findings might suggest that IGF2 plays an important role in growth and glycolipid metabolism during both pregnancy and postnatal development.

## Introduction

Gestational diabetes mellitus (GDM) is a common complication during pregnancy, and macrosomia is one of the major adverse pregnancy outcomes of GDM (1). Insulin-like growth factor 2 (IGF2) encodes a polypeptide that is abundant in fetal tissues and circulation (2), is usually expressed in fetal tissue and invasive trophoblast cells at the placental maternal-fetal interface (3) and is the major insulin-like growth factor that plays a growth-promoting role during the process of embryonic development (4, 5). It has been reported that IGF2 regulates nutrient supplementation by the placenta, and its level in the fetal circulation reflects the growth rate of fetal tissues and nutrient demand (2, 6). The IGF2 concentration in cord serum was found to have a significantly positive effect on both birth length and weight (7). Our previous studies (8, 9) also indicated that a high glucose concentration in the context of GDM increased the expression of IGF2 and its imprinted gene (H19) by changing the levels of methylation in the IGF2 and H19 gene promoters, which might be the underlying pathogenic mechanism of macrosomia.

A study by Street et al. suggested that the fetal environment had long-term effects on growth (10). A mouse study found cases in which fetal IGF2 is misregulated (Beckwith-Wiedemann and Silver-Russell syndromes) can be diagnosed, and growth can be rescued by prenatally adjusting IGF2 or its signaling pathway (11). Compared to normal-weight children, IGF2 concentrations are increased in obese children (12). IGF2 expression in the blood shows an upward trend with increasing body mass index (BMI) in women (13). One study (14) demonstrated that children with catch-up growth in the first two years after birth had higher levels of IGF2. The IGF2 level at age five was closely correlated with five-year-old fat mass as well as the IGF2 level at birth (14). Therefore, IGF2 is also expressed postnatally in humans and may play an important role in growth and development.

IGF2 can combine with several IGF/insulin (INS) receptors (IGF1R, INSR, IGF1/INSR hybrids and IGF2R) to exert autocrine, paracrine and endocrine effects (15, 16). A case report (17) showed that patients with tumors that overproduced IGF2 developed hypoglycemia. IGF2, similar to insulin, can promote hypoglycemia by enhancing glucose uptake by skeletal muscle and inhibiting glucose release from the liver (16). At the same time, IGF2 can also suppress free fatty acid release and glucagon secretion (16).

To investigate the effects of IGF2 on mouse weight gain and glycolipid metabolism, as well as the underlying mechanism, we constructed a mouse model of IGF2 overexpression to record weight gain before adulthood, to obtain the values of adult glycolipid metabolism indicators in the peripheral blood and to detect the expression of genes in the IGF2 signaling pathway in different mouse tissues. In addition, we explored the independent association between IGF2 gene expression and macrosomia by comparing the expression levels of IGF2 mRNA/H19 RNA in maternal peripheral blood and fetal cord blood of humans.

## Materials and methods

### Mouse model of IGF2 gene overexpression

**Figure 1:**
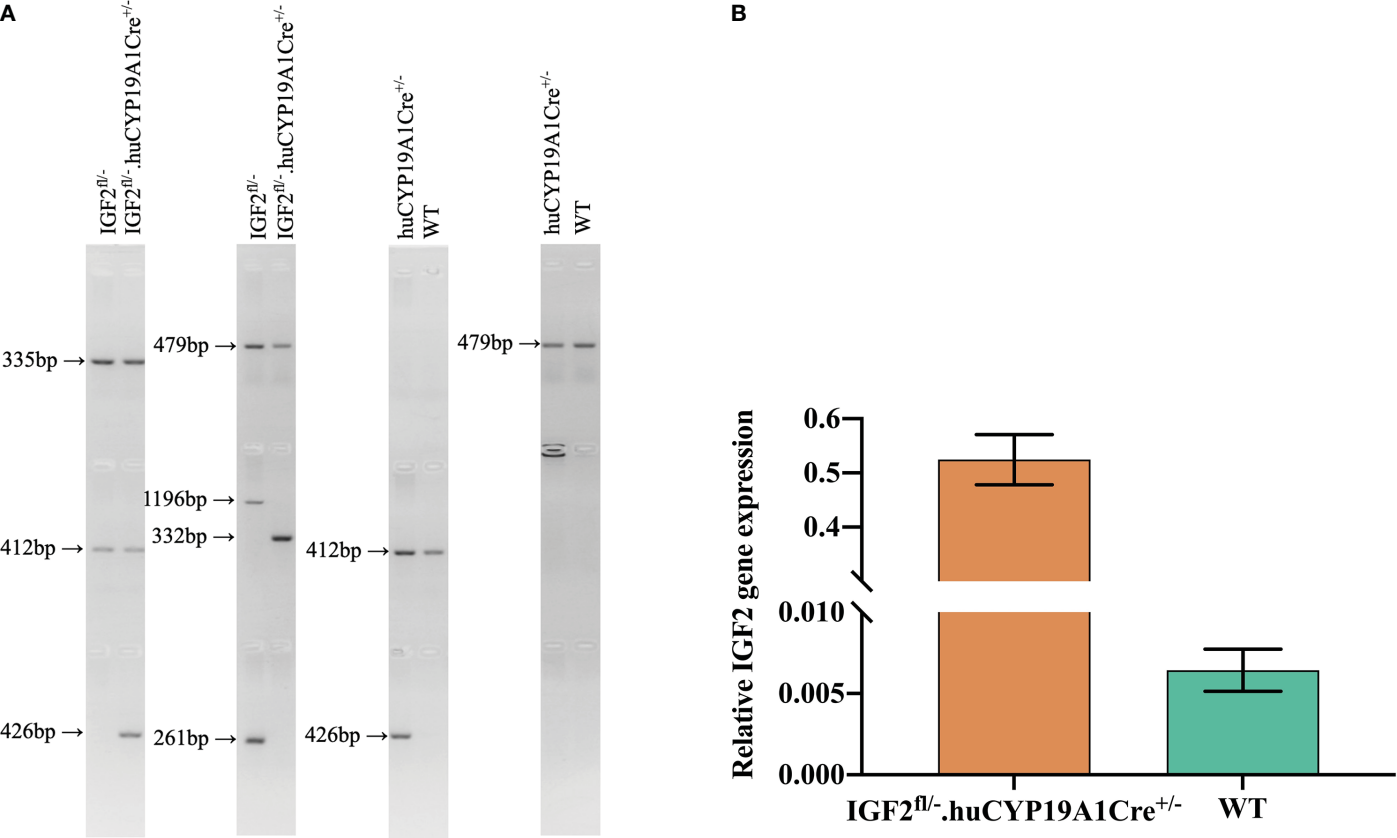
Identification of targeted mice with IGF2 gene overexpression. **(A)** Mouse genotyping by agarose gel PCR electrophoresis with the primers in **Table 1**. **(B)** Real-time quantitative PCR of the IGF2 gene in the livers of IGF2^fl/-^. huCYP19A1Cre^+/-^ and WT mice (n=5-6 mice in each group).

IGF2^fl/fl^ or IGF2^fl/-^ female mice were obtained by site-directed insertion of a murine IGF2 gene fragment containing the Loxp structure into H11 of C57BL/6 mice using the CRISPR/Cas9 strategy. At the same time, the CRISPR/Cas9 strategy was also used to obtain huCYP19A1Cre^+/-^ male mice by inserting a cyclization recombinase (Cre) gene fragment with the promoter of huCYP19A1 (human cytochrome P450 family 19 subfamily A member 1) into Rosa26 of C57BL/6 mice. Female IGF2^fl/fl^ or IGF2^fl/-^ mice were mated with male huCYP19A1Cre^+/-^ mice to obtain four genotypes of offspring, IGF2^fl/-^. huCYP19A1Cre^+/-^, IGF2^fl/-^, huCYP19A1Cre^+/-^ and wild type (WT). Offspring mice with both the exogenously inserted IGF2 gene and the Cre gene fragment exhibit overexpression of IGF2. The offspring mice were marked by toe clipping within one week after birth. Samples of the mouse toes were used for genotyping by agarose gel PCR electrophoresis (**Figure 1A**). The relevant primer sequences are shown in **Table 1**. The following primer was used in the real-time quantitative PCR of IGF2 gene in the livers of IGF2^fl/-^. huCYP19A1Cre^+/-^ and WT mice (**Figure 1B**): sense 5’-GGAGCTTGTTGACACGCTTCAGT-3’ and antisense 5’-GAAGCAGCACTCTTCCACGATG-3’. Mice in this study were manipulated according to the principles of laboratory care and approved by the Laboratory Animal Welfare Ethics Committee of Peking University First Hospital (No. J201941).

**Table 1:** Primers for gene identification in the mouse model of IGF2 gene overexpression.

Purpose		Forward	Reverse	Expected band size
Exogenous IGF2	CKI	GGGCAGTCTGGTACTTCCAAGCT	TGGCGTTACTATGGGAACATACGTC	335 bp
	WT	CAGCAAAACCTGGCTGTGGATC	ATGAGCCACCATGTGGGTGTC	412 bp
Cre	KI	CCTGCTGTCCATTCCTTATTCCATA	TCGGGTGAGCATGTCTTTAATCT	426 bp
	WT	CCCAAAGTCGCTCTGAGTTGTTA	TCGGGTGAGCATGTCTTTAATCT	479 bp
Effect of Cre	Pre-Cre	CTAGAGCCTCTGCTAACCATGTTC	CGGTCCGAACAGACAAACTGAAG	1196 bp
	Post-Cre			332 bp
	Pre-Cre	TCCCCATCAAGCTGATCCGG	CGGTCCGAACAGACAAACTGAAG	261 bp

IGF2, insulin-like growth factor 2; Cre, cyclization recombinase; CKI, conditional knock-in; WT, wild type; KI, knock-in; Post-Cre, after the effect of Cre was exerted; Pre-Cre, before the effect of Cre was exerted.

### Recording of mouse weight by week

Pregnant female IGF2^fl/fl^ or IGF2^fl/-^ mice were observed from the 20th day of gestation to confirm delivery. The day that offspring were found was defined as day one. The offspring mice were marked by toe clipping within one week after birth. All offspring mice were weaned at 3 weeks old and fed sterile maintenance feed for mice and rats (SPF biotechnology, China) at 3-10 weeks of age. The weights of the mice were measured and recorded at the same time every week.

### Detection of glycolipid metabolism indicators in the peripheral blood of offspring mice

Ten-week-old offspring mice were anesthetized (sodium pentobarbital, 150-200 mg/kg) to collect blood by removing one of the eyeballs, and the serum was separated out. Glucose concentration, total cholesterol, triglyceride, high-density lipoprotein cholesterol (HDL-C) and low-density lipoprotein cholesterol (LDL-C) were detected by the glucose oxidase method, cholesterol oxidase-peroxidase (CHOD-PAP) method, glycerol phosphate oxidase-peroxidase (GPO-PAP) method, direct selective inhibition method and direct protective reagent method, respectively. An iodine [^125^I] insulin radioimmunoassay kit was used to detect insulin and insulin antibodies.

### Real-time quantitative PCR of genes in the IGF2 signaling pathway in adult mouse tissues

**Figure 2:**
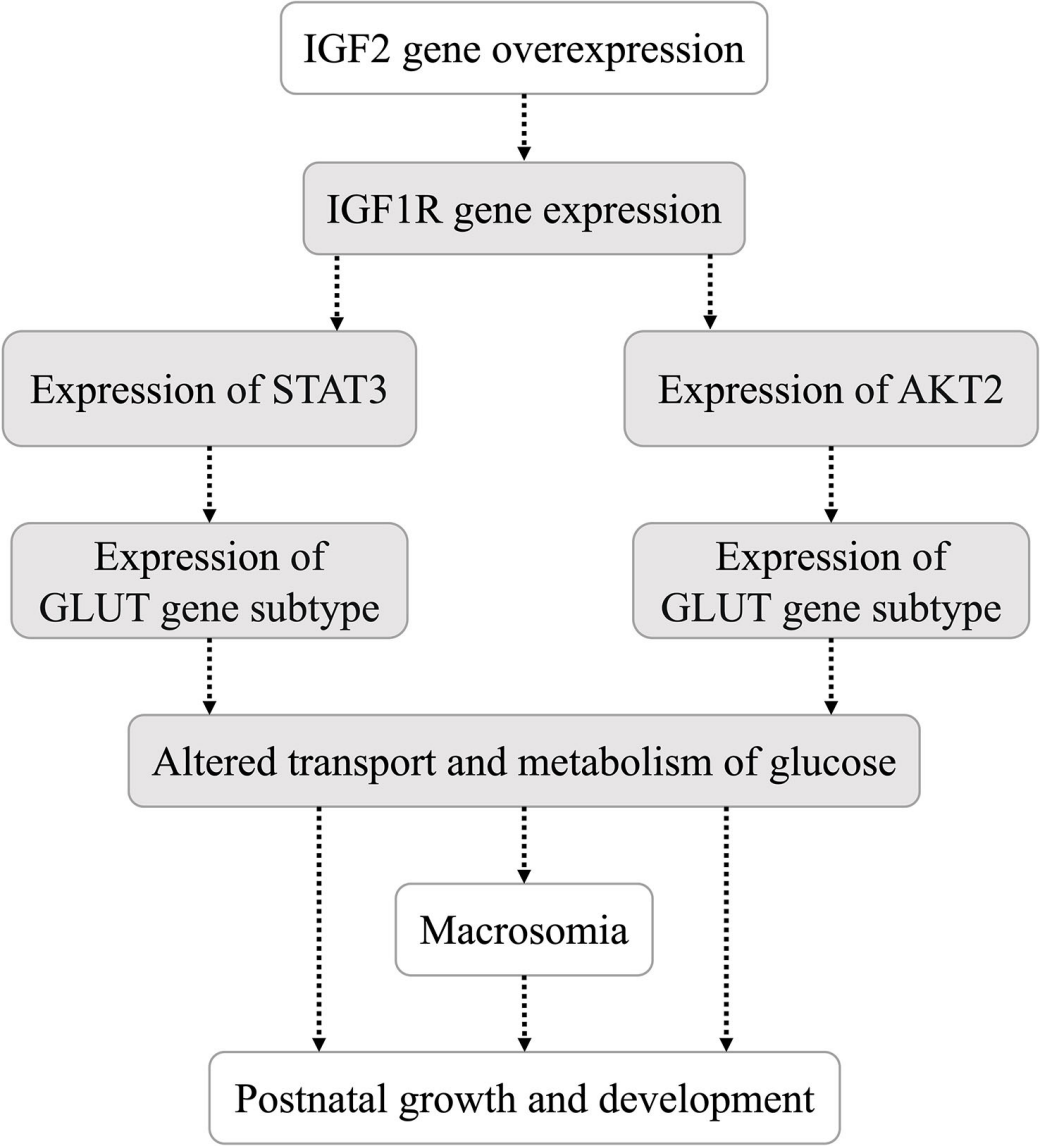
Hypothesis of the IGF2 gene downstream signaling pathway in this study.

The liver, pancreas, kidney, muscle and adipose tissues of 10-week-old offspring mice were collected into liquid nitrogen immediately after anesthesia and blood collection. An appropriate amount of tissue was placed in 1 ml TRIzol reagent and ground with a mixed refrigerated ball mill (Retsch MM400, Germany) at 30 Hz for 6 min to extract total RNA. A Nanodrop 2000 ultramicro-spectrophotometer (Thermo, USA) was used to measure the concentration of extracted RNA. Two micrograms of total RNA were used as the template to synthesize cDNA by reverse transcription, and the cDNA sample was diluted 5 times. Real-time quantitative PCR was performed with a 20 μl reaction system (including 10 μl of Powerup SYBR green master mix, 2 μl of primers, 1 μl of cDNA and 7 μl of RNase-free water). The primer sequences (18–20) used for real-time quantitative PCR of genes in the IGF2 signaling pathway (**Figure 2**) are shown in **Table 2**. The internal reference gene was glyceraldehyde phosphate dehydrogenase (GAPDH).

**Table 2:** Primers for real-time quantitative PCR of mouse tissues.

Gene	Forward	Reverse
H19	GGTGTCTCGAAGAGCTCGGA	CCATGGTGTTCAAGAAGGCTGG
IGF1R	ACTGACCTCATGCGCATGTGCTGG	CTCGTTCTTGCGGCCCCCGTTCAT
STAT3	CAATACCATTGACCTGCCGAT	GAGCGACTCAAACTGCCCT
AKT2	TGACTATGGGCGAGCAGTGG	CTCCATGACCTCCTTCGCATC
GLUT2	TGGAAGGATCAAAGCAATGTTG	CATCAAGAGGGCTCCAGTCAA
GLUT4	CGTTGGTCTCGGTGCTCTTAGTA	GCAGAGCCACGGTCATCAAG
GAPDH	AGGTCGGTGTGAACGGATTTG	GGGGTCGTTGATGGCAACA

IGF1R, insulin-like growth factor 1 receptor; AKT2, AKT serine/threonine kinase 2; STAT3, signal transducer and activator of transcription 3; GLUT2, glucose transporter 2; GLUT4, glucose transporter 4; GAPDH, glyceraldehyde phosphate dehydrogenase.

### Real-time quantitative PCR of the IGF2/H19 gene in maternal peripheral blood and fetal cord blood

A total of 26 healthy pregnant women were involved in this study, and patients with complications were excluded (including but not limited to multiple gestation, GDM, pregnancy-induced hypertension, premature birth, and fetal anomalies). Macrosomia was defined as a fetus with a birth weight > 4000 g. Blood samples from 26 pregnant women were classified into four groups based on whether the neonates had macrosomia or normal birth weight: maternal peripheral blood of macrosomia (MM, n=14), fetal cord blood of macrosomia (MF, n=14), maternal peripheral blood of neonates with normal birth weight (CTRL-M, n=12), and fetal cord blood of neonates with normal birth weight (CTRL-F, n=12). Total RNA was extracted from the blood samples and used to synthesize cDNA by reverse transcription. Then, the cDNA was used in a 480II real-time quantitative PCR system (Roche, Switzerland) to detect the expression levels of IGF2 and H19 in each group of blood samples, and the sequences of the primers (21) used are shown in **Table 3**. The present study was approved by the Ethics Committee of Peking University First Hospital (No. 2013-572). All participants who provided blood samples signed written informed consent forms.

**Table 3:** Primers for real-time quantitative PCR of human blood.

Gene	Forward	Reverse
IGF2	TGGACACCCTCCAGTTCGTC	GCGGAAACAGCACTCCTCAA
H19	ACTCAGGAATCGGCTCTGGAA	CTGCTGTTCCGATGGTGTCTT
GAPDH	GAAGGTGAAGGTCGGAGTC	GAAGATGGTGATGGGATTTC

IGF2, insulin-like growth factor 2; GAPDH, glyceraldehyde phosphate dehydrogenase.

### Statistical analysis

SPSS Statistics 26.0 (IBM, USA) and GraphPad Prism 9 (GraphPad, CA) were used to analyze the data in this study. Data were tested by the D’Agostino & Pearson test to confirm normality. Based on whether the data conformed to a normal distribution, Student’s *t* test or an independent-sample nonparametric test was used for statistical comparisons. The data for comparisons were derived from n ≥ 3 repetitions. *P* < 0.05 indicated that the differences were statistically significant.

## Results

### Overexpression of IGF2 affects mouse weight at 5-10 weeks of age

Among the offspring of the four genotypes (IGF2^fl/-^. huCYP19A1Cre^+/-^, IGF2^fl/-^, huCYP19A1Cre^+/-^ and WT), only the offspring mice with both the exogenously inserted IGF2 gene and the Cre gene fragment (IGF2^fl/-^. huCYP19A1Cre^+/-^) exhibit overexpression of IGF2. The weights of male IGF2^fl/-^. huCYP19A1Cre^+/-^ mice were significantly higher than those of IGF2^fl/-^, WT and huCYP19A1Cre^+/-^ mice at the ages of 5 weeks, 6 weeks and 7/9-10 weeks, respectively (all *P* < 0.05). Compared with IGF2^fl/-^, huCYP19A1Cre^+/-^ and WT mice, female IGF2^fl/-^. huCYP19A1Cre^+/-^ mice exhibited significant increases in body weight in mice aged 5-10 weeks (all *P* < 0.05). Regardless of sex, there was no significant difference in body weight among IGF2^fl/-^, huCYP19A1Cre^+/-^ and WT mice from 5-10 weeks of age. The mouse weights are shown in **Table 4**.

**Table 4:** Comparison of mouse weights at 5-10 weeks old across four genotypes.

Age (week)		IGF2^fl/-^.huCYP19A1Cre^+/-^(Weight, g)	IGF2fl/- (Weight, g)	huCYP19A1Cre^+/-^ (Weight, g)	WT (Weight, g)
Male	5	21.6 ± 1.7^a^	18.3 ± 2.3	18.3 ± 2.7	19.1 ± 1.1
	6	22.4 ± 1.5^c^	21.1 ± 1.6	21.1 ± 0.8	20.6 ± 1.0
	7	23.8 ± 1.5^b^	23.0 ± 1.5	22.6 ± 0.8	22.6 ± 1.2
	8	24.9 ± 1.2	23.8 ± 1.2	23.8 ± 0.7	23.7 ± 1.0
	9	26.0 ± 1.3^b^	25.1 ± 1.7	24.0 ± 0.9	24.9 ± 1.1
	10	26.6 ± 1.4^b^	26.0 ± 1.6	25.0 ± 1.1	25.7 ± 1.2
Female	5	18.5 ± 1.1^abc^	15.8 ± 2.4	15.8 ± 1.1	16.7 ± 1.1
	6	20.0 ± 1.0^abc^	17.5 ± 1.2	16.7 ± 1.1	17.7 ± 1.1
	7	20.7 ± 1.4^abc^	18.2 ± 1.1	17.7 ± 1.0	18.6 ± 1.1
	8	21.4 ± 1.2^abc^	18.7 ± 0.9	18.7 ± 0.9	19.2 ± 0.8
	9	21.9 ± 1.4^abc^	19.5 ± 1.1	19.6 ± 1.0	19.9 ± 0.9
	10	22.7 ± 1.2^abc^	20.2 ± 1.1	20.0 ± 0.7	20.4 ± 0.9

WT, wild type. ^a^Compared with IGF2^fl/-^ mice, P < 0.05; ^b^Compared with huCYP19A1Cre^+/-^ mice, P < 0.05; ^c^Compared with WT mice, P < 0.05; n=7-17 mice in each group.

### Overexpression of IGF2 affects glycolipid metabolism in adult mice

**Figure 3:**
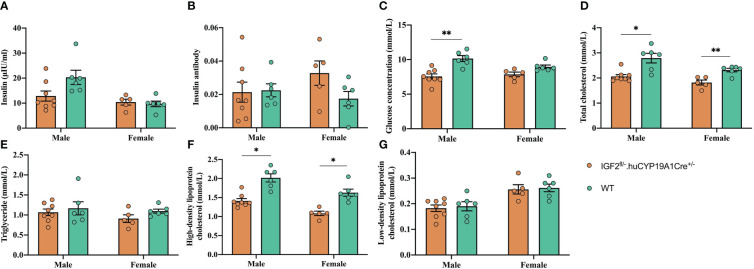
Effect of IGF2 overexpression on glycolipid metabolism in adult mice. The insulin **(A)**, insulin antibody **(B)**, glucose concentration **(C)**, total cholesterol **(D)**, triglyceride **(E)**, high-density lipoprotein cholesterol **(F)** and low-density lipoprotein cholesterol **(G)** levels of adult IGF2^fl/-^. huCYP19A1Cre^+/-^ and WT mice; n=5-8 mice in each group. ^*^ *P* < 0.05; ^**^ *P* < 0.01.

The glucose concentration, total cholesterol and HDL-C levels of IGF2^fl/-^. huCYP19A1Cre^+/-^ male mice were significantly lower than those of WT mice (all *P* < 0.05). The levels of total cholesterol and HDL-C in female IGF2-overexpressing mice (IGF2^fl/-^. huCYP19A1Cre^+/-^) were significantly lower than those in WT female mice (all *P* < 0.05). In addition, overexpression of IGF2 had no significant effect on the levels of insulin, insulin antibodies and triglycerides, regardless of mouse sex. The results are shown in **Figure 3**.

### Overexpression of IGF2 affects gene expression of the glycolipid metabolism pathway in adult mouse tissues

**Figure 4:**
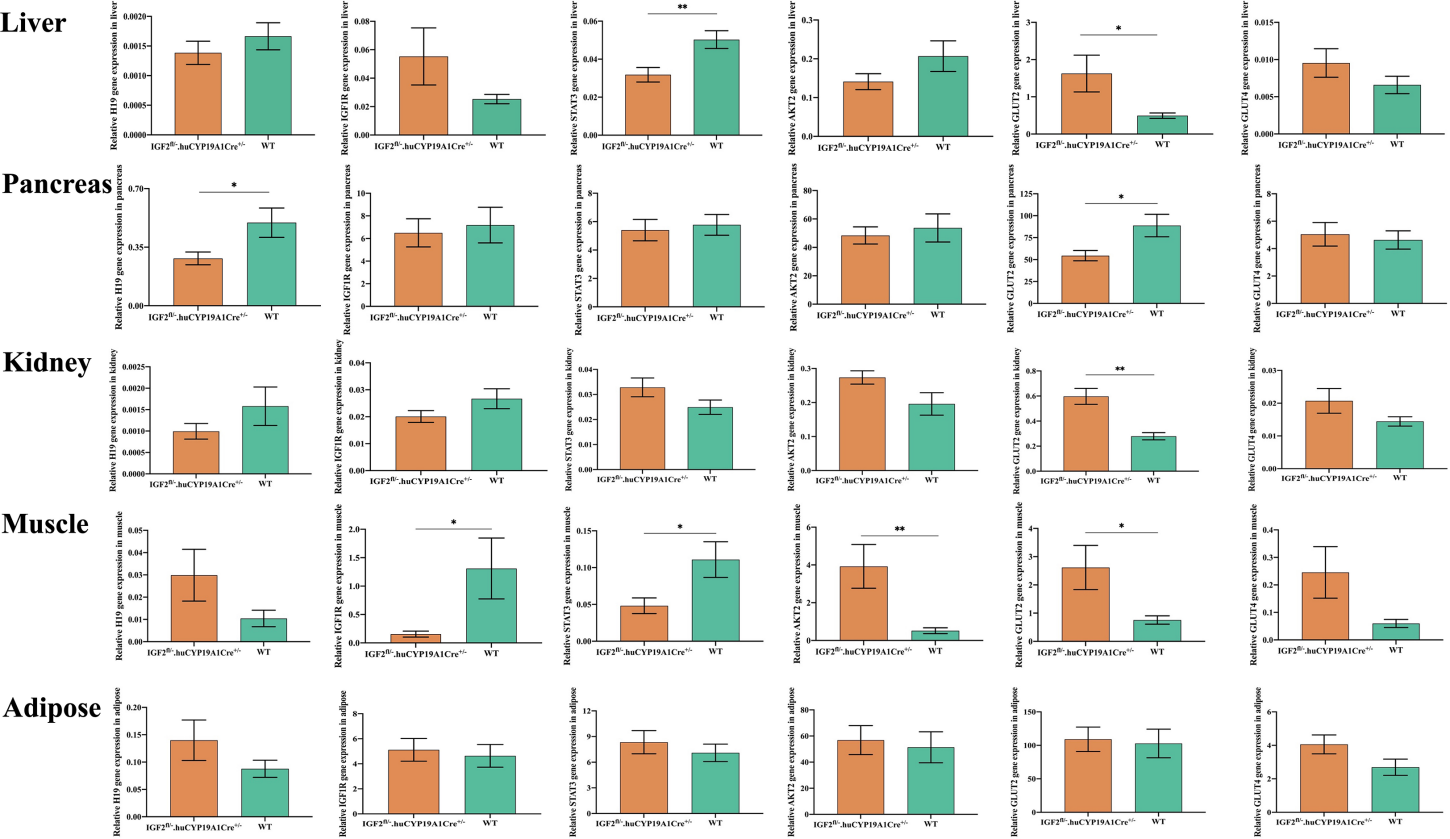
Effects of IGF2 overexpression on the genes of the pathway related to glycolipid metabolism in adult mouse tissues; n=4-6 mice in each group. ^*^ *P* < 0.05; ^**^ *P* < 0.01.

It was reported (16) that IGF2, similar to insulin, can promote hypoglycemia by enhancing glucose uptake by skeletal muscle and inhibiting glucose release from the liver. In addition, IGF2 can suppress free fatty acid release from adipose tissue and glucagon secretion from the pancreas (16). As shown in **Figure 4**, compared with that of WT mice, the expression of H19 was significantly decreased in the pancreas and IGF1R was significantly decreased in the muscle of mice with IGF2 overexpression (IGF2^fl/-^. huCYP19A1Cre^+/-^). The signal transducer and activator of transcription 3 (STAT3)/AKT serine/threonine kinase (AKT) axis is a classic downstream pathway of the IGF/IGF1R signaling pathway (22). The expression levels of STAT3 and AKT2 showed significant decrease in liver, muscle and increase in muscle of IGF2-overexpressing mice, respectively. At the same time, as key molecules involved in glucose metabolism, the expression of glucose transporter (GLUT) 2 and GLUT4 also showed alterations in IGF2-overexpressing mice. Notably, GLUT2 expression showed significant increase in liver, kidney, muscle and decrease in pancreas of mice with IGF2 overexpression, suggesting that it might be an important part of the pathway downstream of IGF2.

### Independent association between IGF2 expression and macrosomia

As shown in **Table 5**, the neonatal birth weights [mean (standard deviation)] in the macrosomia and normal birth weight groups were 4192 (165) g and 3404 (299) g, respectively, which were significantly different (*P* < 0.01). Maternal age, height, gestation age, prepregnancy weight, predelivery weight and gestational weight gain showed no statistically significant difference between the macrosomia and normal birth weight groups.

**Table 5:** Clinical information for pregnant women who provided human blood.

	Macrosomia group (*n* =14)	Normal birth weight group (*n* =12)	*P* value
Age (year)	34.9 ± 5.0	33.2 ± 3.5	0.316
Height (cm)	161.4 ± 4.6	164.6 ± 5.7	0.120
Gestation age (wk)	39.6 ± 1.1	39.2 ± 0.7	0.215
Neonatal birth weight (g)	4192 ± 165	3404 ± 299	< 0.01
Prepregnancy weight (kg)	58.6 ± 7.7	57.7 ± 4.9	0.721
Predelivery weight (kg)	72.1 ± 8.4	71.9 ± 5.9	0.950
Gestational weight gain (kg)	13.5 ± 4.0	14.2 ± 2.4	0.576

BMI, body mass index.

**Figure 5:**
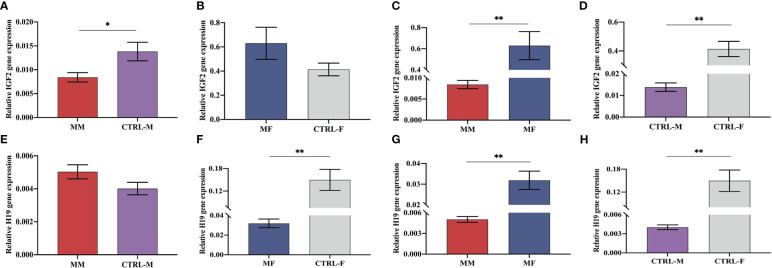
Expression levels of IGF2 mRNA **(A–D)** and H19 RNA **(E–H)** in maternal peripheral blood and the corresponding fetal cord blood of the macrosomia group (MM, MF) and normal birth weight group (CTRL-M, CTRL-F). *P < 0.05; **P < 0.01.

As shown in **Figure 5**, the mRNA expression of IGF2 showed significant decrease and the H19 RNA showed higher expression in the maternal peripheral blood of the macrosomia group (MM), compared with that in the normal birth weight group (CTRL-M) (**Figures 5A, E**). Compared with that in the fetal cord blood of the normal birth weight group (CTRL-F), the mRNA expression of IGF2 in the fetal cord blood of the macrosomia group (MF) was increased (**Figure 5B**), and the RNA expression of H19 was significantly reduced (**Figure 5F**). Comparisons of gene expression in the maternal peripheral blood and corresponding cord blood of the same group showed that IGF2 expression in the cord blood was significantly higher than that in the maternal peripheral blood (**Figure 5C**), and the fold difference was higher in the macrosomia group than that in the normal birth weight group (**Figures 5C, D**). In addition, the RNA expression of H19 showed the opposite trend (**Figures 5G, H**). These results might indicate that in the case of normal mothers with similar clinical characteristics, IGF2 expression is an independent factor influencing macrosomia.

## Discussion

By constructing a mouse model of IGF2 overexpression, we showed in the present study that IGF2 overexpression significantly increased the weights of mice at 5-10 weeks of age [equivalent to human puberty, which corresponds to 12-18 years of age (23)] and changed the values of adult glycolipid metabolism indicators, which might be the result of changes in the IGF1R-STAT3/AKT2-GLUT2/GLUT4 downstream pathway of IGF2. In addition, we found an independent correlation between IGF2 levels and macrosomia by comparing the expression levels of IGF2/H19 in maternal peripheral blood and fetal cord blood.

A previous study suggested that IGF2 levels at age five were closely correlated with five-year-old fat mass, and children with catch-up growth in the first two postnatal years had higher levels of IGF2 (14), which indicated that IGF2 might play an important role in the postnatal growth and development of humans. The results of this study showed that compared with WT siblings, mice with IGF2 overexpression showed significant increase in weight at 5-10 weeks of age. Previous studies (14, 24) reported that a sex bias exists in the expression of IGF2, and the IGF2 level in females is significantly higher than that in males at the age of 5 years. Moreover, another study (25) found that hypoxic conditions lead to the upregulation of IGF2 expression in the placenta of female fetuses, while the hypoxic placenta of male fetuses shows no change. In the present study, the weight increases at the age of 5-10 weeks induced by IGF2 overexpression in female mice were more significant than those in male mice. In addition, a previous study (13) evaluated blood samples from women aged 40-79 years and found that IGF2 shows an upward trend in blood as BMI increases, which means that IGF2 may also have an important effect on weight changes during and after adulthood.

Our previous study (8) demonstrated that high glucose concentrations can upregulate the expression of IGF2. However, excessive expression of IGF2 can result in the occurrence of hypoglycemia (17). The present study showed that the glucose concentration in the blood of mice with IGF2 overexpression was significantly lower than that in the blood of WT mice (**Figure 3C**). Therefore, the upregulation of IGF2 in response to high-glucose conditions may be a feedback loop to increase the utilization of glucose and reduce the blood glucose level, which probably contributes to weight gain. A previous paper (26) indicated that male offspring of GDM mice have lower expression of IGF2 in the liver, and they show more severe glucose intolerance and insulin resistance than female offspring. Elevated IGF2 levels may be beneficial in stabilizing blood glucose concentrations, such as in the intrauterine hyperglycemic environment in women with GDM, but the consequence of this process affecting weight gain probably leads to macrosomia, which is an adverse pregnancy outcome. In addition, IGF2 also has the ability to reduce lipolysis and inhibit free fatty acids (16). The results of this study showed that total cholesterol and HDL-C were significantly reduced in mice with IGF2 overexpression. The interaction between IGF2 and lipid metabolism needs to be further studied.

IGF2 can combine with several IGF/insulin (INS) receptors (IGF1R, INSR, IGF1/INSR hybrids and IGF2R) to exert autocrine, paracrine and endocrine effects (15, 16). The STAT3/AKT axis is a typical downstream pathway of the IGF/IGF1R signaling pathway (22). Both STAT3 and AKT are upstream regulators of glucose transporters (27, 28). In the present study, the expression levels of underlying downstream genes (including H19, IGF1R, STAT3, AKT2, GLUT2 and GLUT4) of IGF2 were analyzed in multiple organs and tissues of mice with IGF2 overexpression. IGF2 overexpression induced altered expression of downstream genes. The hypothesis that IGF2 regulates the transport and metabolism of glucose through the IGF1R-STAT3/AKT2-GLUT2/GLUT4 pathway was preliminarily investigated. Due to the different effects of IGF2 overexpression on tissues involved in glycolipid production, storage or consumption, the specific process by which IGF2 overexpression regulates its downstream pathway in each tissue needs to be explored further.

Based on previous studies, it was determined that IGF1 has high postnatal expression (29, 30) and that the expression of IGF2 is high during embryonic development but decreases to an undetectable level after birth (30–32). Several recent studies (12–14, 24, 33) have suggested that IGF2 is also expressed postnatally in humans and may play an important role in growth and development. Due to the previous opinion, there is currently a lack of related research on human postnatal IGF2 compared to studies on postnatal IGF1 (24). The present study constructed a mouse model of IGF2 overexpression, whose postnatal level of IGF2 was higher than that of WT mice, and the effects of IGF2 overexpression on the postnatal growth and glycolipid metabolism of the mice were explored, which might provide evidence for the postnatal effects of IGF2. However, this study also had some limitations. The effects of IGF2 overexpression on the birth weights and prefive-week-old weights of offspring mice were not significant, which might be the result of the influence of different litter sizes (34, 35). Therefore, to compensate for the deficiency of the mouse model, we used maternal peripheral blood and fetal cord blood samples from normal singleton pregnancies to demonstrate the independent correlation between IGF2 expression and macrosomia.

It was reported that IGF2 cord serum concentration had a significant positive effect on both birth length and weight (7) and IGF2 content in the placental lysates was one of the most important factors associated with fetal growth restriction (36). Our previous study (9) indicated that in normal pregnant women, the expression of IGF2 in the placenta and cord blood was significantly higher for women with macrosomia than for women with normal birth weight neonates. However, the maternal prepregnancy and predelivery BMIs of women with macrosomia were significantly higher than those of women with normal birth weight neonates (9). By controlling for maternal age, height, gestation age, prepregnancy weight, predelivery weight and gestational weight gain, the present study demonstrated that IGF2 expression was an independent factor connected with macrosomia.

In conclusion, by constructing a mouse model of IGF2 overexpression, we showed that IGF2 expression was independently correlated with the occurrence of macrosomia, and overexpression of IGF2 significantly increased the weights of mice at the age of 5-10 weeks and significantly affected the values of adult glycolipid metabolism indicators, which might be the result of changes in the IGF2-IGF1R-STAT3/AKT2-GLUT2/GLUT4 pathway. These findings might suggest that IGF2 plays an important role in growth and metabolism during both pregnancy and postnatal development.

## Data availability statement

The raw data supporting the conclusions of this article will be made available by the authors, without undue reservation.

## Ethics statement

The studies involving human participants were reviewed and approved by the Ethics Committee of Peking University First Hospital. The patients/participants provided their written informed consent to participate in this study. The animal study was reviewed and approved by the Laboratory Animal Welfare Ethics Committee of Peking University First Hospital.

## Author contributions

This study was conceived and designed by YW. The data were collected by QZ, SQ and JH with material and technical support from HY. The data were analyzed by QZ and YW. The paper was written by QZ and YW. All authors contributed to the article and approved the submitted version.
